# Supplementary material for: FLIM quality metric visualization as a means to validate consistency across large-area non-homogeneous FLIM datasets
Source: Methods Appl Fluoresc. Author manuscript; Available in PMC 2026 May 13. (PMC13169974; doi:10.1088/2050-6120/ae4e7b)
Supplement: Supplementary file 1 [file NIHMS2166478-supplement-Supplementary_data.pdf]

Export citation and abstract BibTeX RIS

← **Previous** article in issue

Next article in issue →

Original content from this work may be used under the terms of the Creative Commons Attribution 4.0 licence. Any further distribution of this work must maintain attribution to the author(s) and the title of the work, journal citation and DOI.

physicsworld LIVE
in partnership with Reports on Progress in Physics™

Supplementary data

1. Introduction

Fluorescence lifetime imaging microscopy (FLIM) is a powerful tool to study fluorescent samples and probe contrast, using fluorescence decay times to derive information about the fluorophore's environment, supporting its common use as a biological reporter of cell conditions, state, and behavior. FLIM is predominantly deployed in biological sensing applications involving environment-sensitive probes or fluorophores whose decay kinetics are modulated under specific conditions, such as cell membrane tension or molecular binding sensors [1, 2]. However, the technique has also found many applications in intrinsic fluorescence imaging, where endogenous fluorophores provide label-free contrast. For example, FLIM can be used as a biological reporter of metabolism by analyzing molecular binding states of intrinsic fluorophores like NAD(P)H or FAD [3].

Despite the benefits of this technique for biological imaging, FLIM data analysis is often complicated by several sources of variability which can affect the reproducibility of data and complicate downstream analysis. Dataset consistency across sample replicates or imaging sessions can be challenging and often affects post-processing of images. For instance, fluorescence lifetimes are known to be very sensitive to changes in the local environment, such as solvents or temperature, sometimes making it difficult to interpret changes in biological samples [4]. Lifetime measurements are also sensitive to changes in image collection parameters, notably the number of photons per decay curve, which can affect separability of fluorophore species [5, 6]. It is often incorrectly assumed that an intensity image with sufficient contrast will have enough photons to effectively calculate a good FLIM image. Decay curves with low photon count can be compensated for with imaging parameters (higher laser power or longer collection times) or spatial binning of the image at the cost of spatial resolution. Best practices for quantitative image analysis normally requires keeping these parameters as consistent as possible throughout a dataset. However, the number of photons provided for the estimation of the fluorescence lifetime can dramatically impact the result, as shown by theoretical estimates of the Cramér-Rao Lower Bound (CRLB) for variability [7, 8]. The CRLB estimates the minimum variance found in a measurement, which for FLIM scales with both photon count and the lifetime itself [9]. In monoexponential, homogeneous lifetime experiments, we have reported that when the number of photons per decay curve are kept relatively constant, an estimate of the higher lifetime value is less precise as denoted by the coefficient of variation (CV) (figure 1) [9]. While these effects can be mitigated by collecting more photons overall, it may not always be practical in biological settings due to photobleaching or live-cell timing constraints. In contrast, for an ideal FLIM system, the variability would be constant at the same photon level, no matter the lifetime value measured. The increase in variability associated with longer lifetimes is separated from noise introduced by fitting routines as this increase in variability is also shown in phasor space. While we have previously correlated this single-component data with the CRLB, the multi-component CRLB has, to our

knowledge, not yet been theoretically modeled. As an intermediate step for understanding the minimum variance in biexponential distributions, here we provide a method of simulating multicomponent data to understand the variability across phasor space and allow researchers to estimate the potential for separability in biological data, including in large field of view datasets.

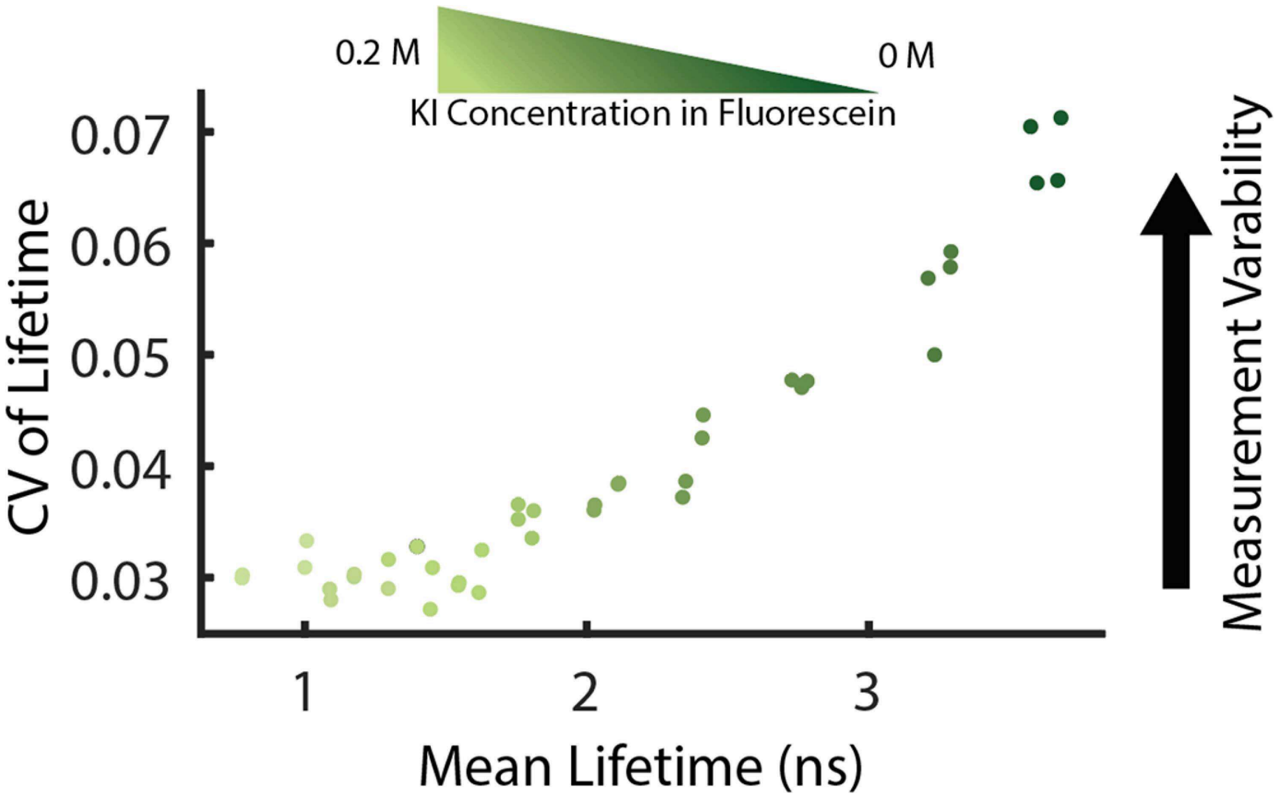

Figure 1. Lifetime imaging of fluorescein dye standards performed with a consistent photon count controlled by laser power adjustments shows despite similar acquisition parameters the coefficient of variation (CV) in lifetime estimates increases at longer lifetime values. Fluorescein dye was quenched with several concentrations of potassium iodide (KI) to create a range of single-component lifetime values [9].

Constructing a viable biological dataset with a consistent signal-to-noise ratio (SNR) can quickly become challenging, even when similar image collection parameters are used [10]. Consistency in reported values and workflows for measurements is still a relevant concern for FLIM imaging [11]. Biological variability compounds with the existing sources of variability in FLIM measurements by introducing biological changes from within (intra-sample variability) and between (inter-sample variability) samples. For example, changes to cellular expression of an intrinsic fluorophore may naturally change the fluorescence intensity for a healthy versus diseased tissue. Normalizing photon count in a sample with autofluorescence is also challenging when local concentrations of different fluorophores would lead to local intensity variation and fluctuations in the SNR across an image. For instance, bright regions of a tissue may limit the maximum laser power able to be used, but a nearby region of interest (ROI) may be dim, resulting in a technically challenging image acquisition and lower than desired photon counts for analysis if other imaging parameters are not adjusted.

SNR values for a FLIM system have been previously defined using the F-value (figure-of-merit), a variability metric which incorporates changes in both signal intensity and lifetime estimation [12–15]. As described by Gerritsen *et al* the F^{-2} value describes the photon economy of a FLIM system [12]. We apply the F-value, denoted as F′ to indicate a new use-case within large-area FLIM data, as a CV metric to help identify biological data with similar SNRs pixel-to-pixel and more confidently compare FLIM data across

and between samples. This CV masking helps researchers determine if the biological ROI is adequately imaged to fit into the selected SNR range. A user interface is provided to make the visualization of variability metrics and image masking easily applied to an imaging workflow.

2. Methods

2.1. Imaging

2.1.1. Sample preparation

Mouse brain tissue samples of proneural (PDGFb) and mesenchymal (NRAS) glioblastoma tumors, induced via a *Sleeping Beauty* transposon-mediated method detailed in Shamsan *et al* were used for large-area FLIM imaging [16]. The background mouse strain was the FVB/NJ WT mice and specifics of the gliomas produced after DNA plasmid injection can be found in previous publications [16–18]. The tissue sections were mounted in paraffin and remained unstained for FLIM imaging. Adjacent sections were stained with hematoxylin and eosin for histological comparison.

Separate aliquots of a 10 μM fluorescein sodium salt (F6377, Sigma-Aldrich, Millipore, MA) stock solution in phosphate-buffered saline (PBS) were quenched with varying amounts of 1 M potassium iodide (KI) (ChemCenter, TX) as previously described [9]. Final concentrations of KI in 1 ml fluorescein ranged from 0–0.4 M to create a range of lifetimes from approximately 1–4 ns. Droplets (50 μl) of each solution were placed on a glass-bottom petri dish or 8-well plates for imaging.

2.1.2. Optical setup

Tissue imaging was performed on a custom-built multiphoton microscope designed for time-correlated single-photon counting (TCSPC) as previously described [19, 20]. The system is based on an inverted Nikon TE2000, equipped with a Titanium: Sapphire excitation laser (80 MHz, 690–1040 nm, MaiTai DeepSee, SpectraPhysics, CA). The light was modulated using an electro-optic modulator (Model 350-80, ConOptics, CT) and raster scanned with a pair of galvanometer scanners (Cambridge Technology, MA). A 680 nm shortpass filter (FF01-680/SP-25, Semrock, NY) was used to remove the excitation light (740 nm), and NAD(P)H emission was collected with a 457 nm bandpass filter (S457/50M, Chroma, VT). The FLIM signal was collected with a photomultiplier tube (PMT, H7422-40, Hamamatsu Corporation, Japan) and recorded using SPC-180 fast timing electronics (Becker & Hickl, Berlin, Germany). The instrument response function (IRF) used to calibrate FLIM analysis was collected by imaging urea crystals with a 370 nm narrow bandpass filter (FF01-370/10-25, Semrock, NY) for 30 frames. For both tissue and the urea crystals, a Nikon 20 × 0.75NA air objective was used, with a field-of-view (FOV) of approximately 187 × 187 μm, pixel size 0.733 μm.

For the long-duration image acquisition, the microscope was controlled with a Pymmcore-plus (https://pymmcore-plus.github.io/pymmcore-plus/) script running MicroManager and lab-built laser scanning control software (OpenScan, https://github.com/openscan-lsm) [21]. The script automated the stage movement and image collection. Each image was collected for 90 frames and tiling was set with a 5% overlap. The z-coordinates were calculated based on a plane estimate, fit to the z-focus of manually selected sample points in the tile grid. If the PMT detector overloaded, the detector was automatically reset before the next image began and the overloaded images were removed from the analysis. The Pockels cell value to control laser power was set at 0 for <10 mW power at the sample, and the PMT gain was 70%.

Briefly, fluorescein sodium salt dye imaging was performed on the same custom-built microscope. Samples were excited with 890 nm and emission wavelengths were filtered with a 650 nm short pass filter (FF01-650/SP-25, Semrock, NY) and a 520/35 nm bandpass filter (FF01-520/35-25, Semrock, NY). Images were collected using a Nikon lambda 40 × 1.25 WI lens, pixel size 0.366 μm, for 45 frames with 5 μs pixel dwell time. Photon count was kept consistent by modulating the laser power, approximately 20–24 photons per pixel before binning. Analysis of the lifetime data was performed in SPCImage (v8.9, Becker & Hickl, Berlin, Germany) using a single-exponential model, spatial bin of 4, and shift of zero after loading the IRF. The IRF was collected by imaging urea crystals at 890 nm using a 445 nm bandpass filter (ET445/30x, Chroma, VT) for 30 frames. Further details are addressed in a previous publication [9].

2.2. Data processing

2.2.1. SPCImage tissue analysis

FLIM analysis was completed with SPCImage software (version 8.9, Becker & Hickl, Berlin, Germany). For tissues, we used biexponential fitting with maximum likelihood estimation and a spatial bin value of three (corresponding to 7 × 7 pixel binning). The shift value was set to 0 for all images after the respective IRF was imported for each tissue.

2.2.2. Fluorescence lifetime analysis

Images were stitched using the Grid/Collection stitching plugin available in Fiji based on a tile configuration file of the stage coordinates written by the acquisition script [22]. Intensity images used linear blending and lifetime images used average blending. Images are either in grayscale, mlp-viridis, mlp-plasma, or 16-colors look up tables (LUTs) from Fiji [22]. The coefficient of variation (CV) map for the lifetime and intensity images, with CV = σ/μ as the ratio of the standard deviation (σ) to the mean (μ), was calculated in python using a kernel size of 49 × 49 (see GitHub: https://github.com/uw-loci/flim-metrics-viewer). A larger kernel size was used to help blur out very sharp changes in pixel-to-pixel intensity within the tissue. The kernel size should be changed based on the desired coarseness of the CV map and the size of relevant biological structures while still accounting for the bin size for decay curve calculation. Notably, the images were thresholded by intensity for background removal using the Triangle method in Fiji with the removed values converted to Not a Number (NaN) to be excluded from the mean and standard deviation calculation as well as excluded from subsequent boxplots. Images where the detector overloaded were excluded from the stitching and left as blank tiles. The F′-value image accounts for dual variation in intensity and lifetime, calculated according to $F' = CV_\tau/CV_I$ where (τ) is mean lifetime and (I) is the intensity per pixel. F-value for a FLIM system evaluation is expected to be >1, however, here we use it as a CV-metric to address heterogeneous pixel variation. Additionally, single photon counting applies a scaling factor to the F′-value which accounts for the detector dead-time, count rate, and the subtractive noise [12] We chose to use the simplified definition of F-value, omitting the scaling factor, to avoid the addition of fitting-based error of pixel-wise estimation of the offset value. An open source live visualization code with a user interface is also provided so the user can apply a threshold mask, compare regional F′-values within a sample, and save binary mask images (see code in GitHub: https://github.com/uw-loci/flim-metrics-viewer).

2.2.3. Multi-component lifetime simulations

Python code (available on GitHub: https://github.com/uw-loci/flim-metrics) was used to simulate multi-component decay curves based on editable parameters: lifetime values (τ_1 and τ_2), the respective fractional components, time period, photons per decay curve, and background. A lifetime curve for each pixel was created through random sampling of an exponential decay function based on given parameters (examples of phasors provided in supplementary figure S1). We use this method to demonstrate expected variability as a function of photon count and long lifetime values including the range of NAD(P)H measurements. Simulated lifetime images were exported and analyzed in GSLab, a MATLAB phasor analysis program, to get phasor coordinates [23]. A median filter of 1 was applied and all data was calibrated to a single exponential decay at 4 ns. Variability of a single image plotted in phasor space is quantified as a combination of the ellipticity of the coordinate data and the area of the convex hull surrounding the points, excluding outliers based on a constant percentile (see figure 2).

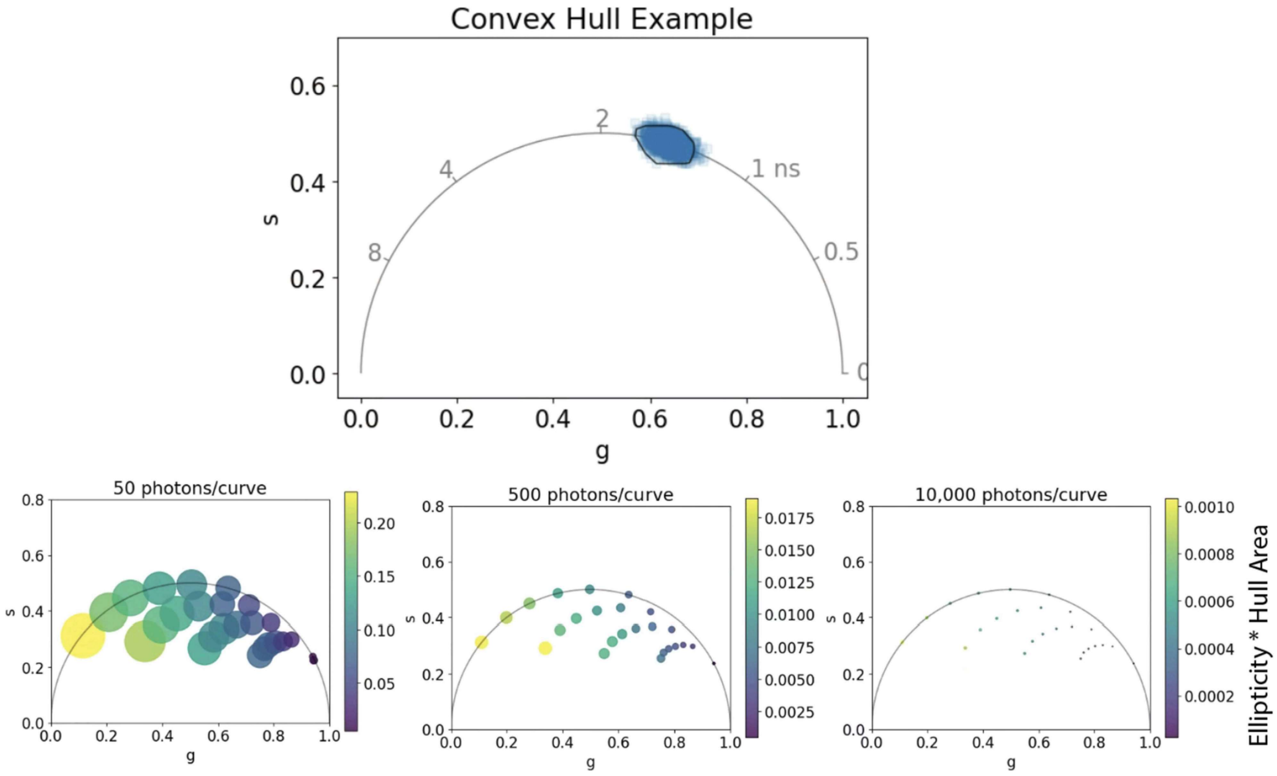

Figure 2. Quantification of variability across phasor space using simulated lifetime data. The phasor plots display multi-component distributions based on a τ_1 of 0.5 ns, τ_2 ranging from 2–8 ns, and fractional components varying from 0–1. The color scale quantifies variability in phasor data using a metric for phasor coordinate area times ellipticity. The size of the circle represents the area of phasor distribution (hull area) scaled by a constant for visualization.

3. Results

3.1. Phasor space variability for multi-component decays

Knowledge of the ground truth lifetime value is necessary for proper characterization of variability. For multi-component decays, simulations are effective for modulating the decay curve and component parameters while still providing values which are reflective of experimental data. Here we simulate biexponential data to represent changes in variability across phasor space, including the range of NAD(P)H lifetimes, by estimating a range of component lifetimes and fractional contribution. Biologically, these ranges can represent changes to cell metabolism or measurements of fluorescent probes. Our results are similar to data shown by Chen *et al* [24]. The spread of coordinate data in phasor space for a single FLIM image depends on background noise, photon count, and the placement of the lifetime in the phasor plot. It is also separated from any error introduced in fitting methods. The phasor variability is quantified by displaying a color map metric based on the area of the convex hull around the coordinate data and the ellipticity value associated with each image (figure 2). The size of the image data point is scaled based on the area of the convex hull, which correlates to the change in photon count and lifetime value. The phasor coordinates in each image become more localized at higher photon counts due to the better estimation of the lifetime. These graphs indicate better fluorophore separation and confidence across all lifetime values when more photons are collected. However, distinguishing shorter lifetimes is easier at lower photon counts due to the reduced spread of phasor coordinates. These simulations can be used to make estimations of experimental data and indicate how many photons will be required to separate fluorophore species in phasor space, leading to better imaging data. This allows researchers to estimate if the SNR of the collected data will have enough phasor space separation to identify distinct populations, even in small sample sizes.

3.2. Intra-sample variability

To study lifetime variability across large-area samples, we have used models of proneural (PDGFb) and mesenchymal (NRAS) glioblastoma tumors, described further by Shamsan *et al* obtained as unstained mouse brain tissue sections mounted in paraffin [16]. We provide adjacent tissue slices stained with hematoxylin and eosin (H&E) as references for the tissue area. Previously, we have demonstrated following select protocols with tissue fixation can conserve trends seen in FLIM data [25]. The use of fixed samples in this study allows for the large-area image collection (more than 1500 image tiles per tissue), which requires continuous imaging over several hours/days. The resolution for a single image tile is displayed in Supplemental figure 2, showing the high pixel-to-pixel heterogeneity in intensity and lifetime within individual images. We also provide calculations of CV maps and F′-value with a 7 × 7 kernel in Supplemental figure 3 to show the change in map coarseness. A larger kernel value (49 × 49) was used in the paper due to the large tissue size. Fixed tissues also reduce the variability effects of imaging in a live or dynamic environment which may have pH or temperature changes, while still being sensitive to the changes in cellular microenvironment across the tissue morphology (intra-sample variability). Despite consistent imaging parameters throughout a continuous acquisition, there is inherent variation across biological samples due to local changes in structure, cell composition, and fluorophore concentration (figure 3). We display a coefficient of variation (CV) map to illustrate the variability across regions of interest (ROIs). These changes in CV highlight areas of the tissue with more or less variability. Differences in lifetime or photon count between images or ROIs can lead to variability of over 15%. If researchers typically sample with small ROIs, these changes may not be apparent in smaller FOVs without a CV calculation. The ROIs and graphs in Supplemental figures 4 and 5 demonstrate the changes in CV for the intensity and mean lifetime across selected tissue areas.

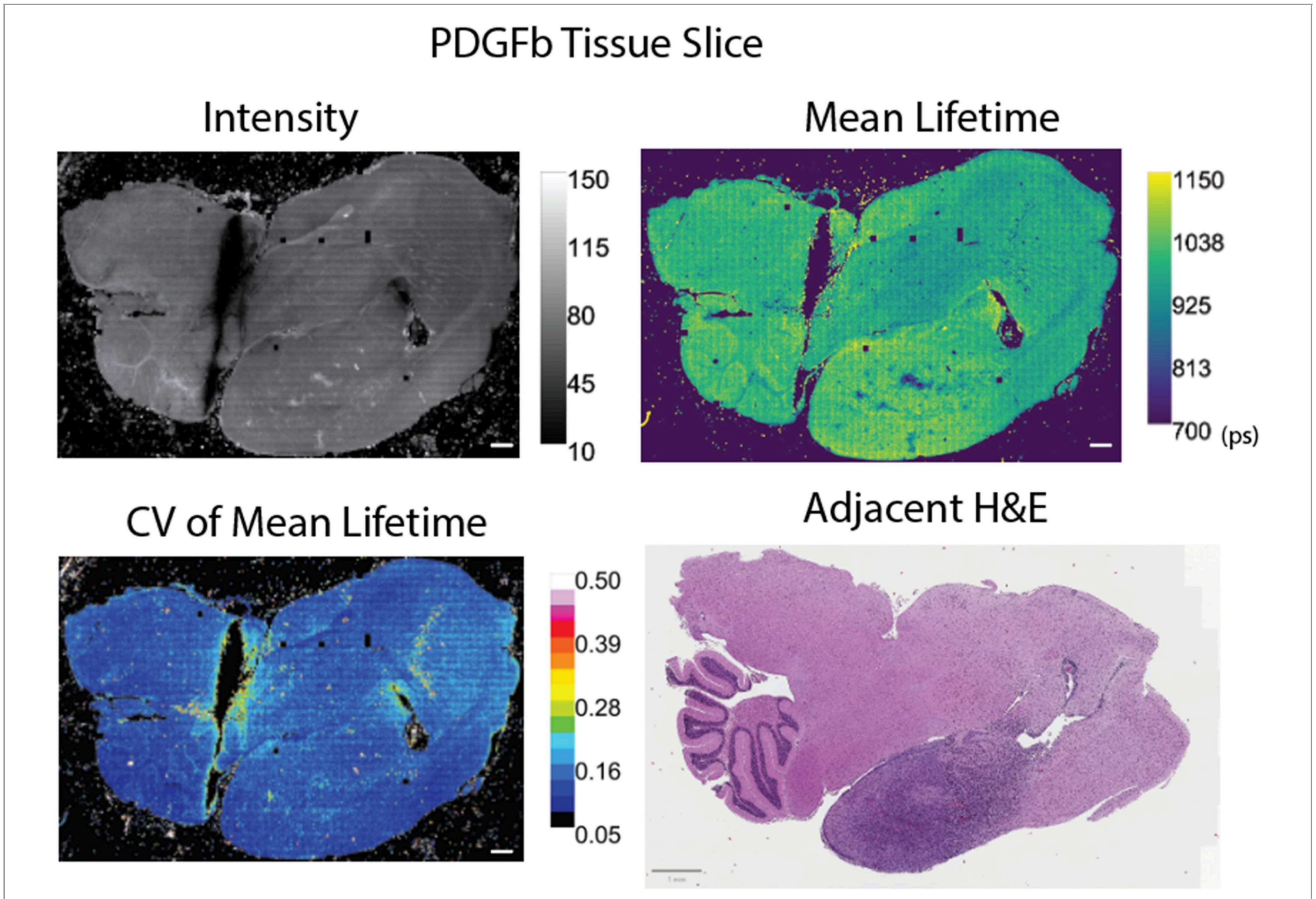

Figure 3. Representative images of PDGFb glioblastoma tissue slice demonstrating intra-sample variability of FLIM signal due to morphology changes and local fluorophore populations: intensity image, mean lifetime (τ) in picoseconds, τ coefficient of variation (CV) map, H&E image of adjacent tissue slice. The scale bar is 1 mm in the H&E and 500 μm

elsewhere.

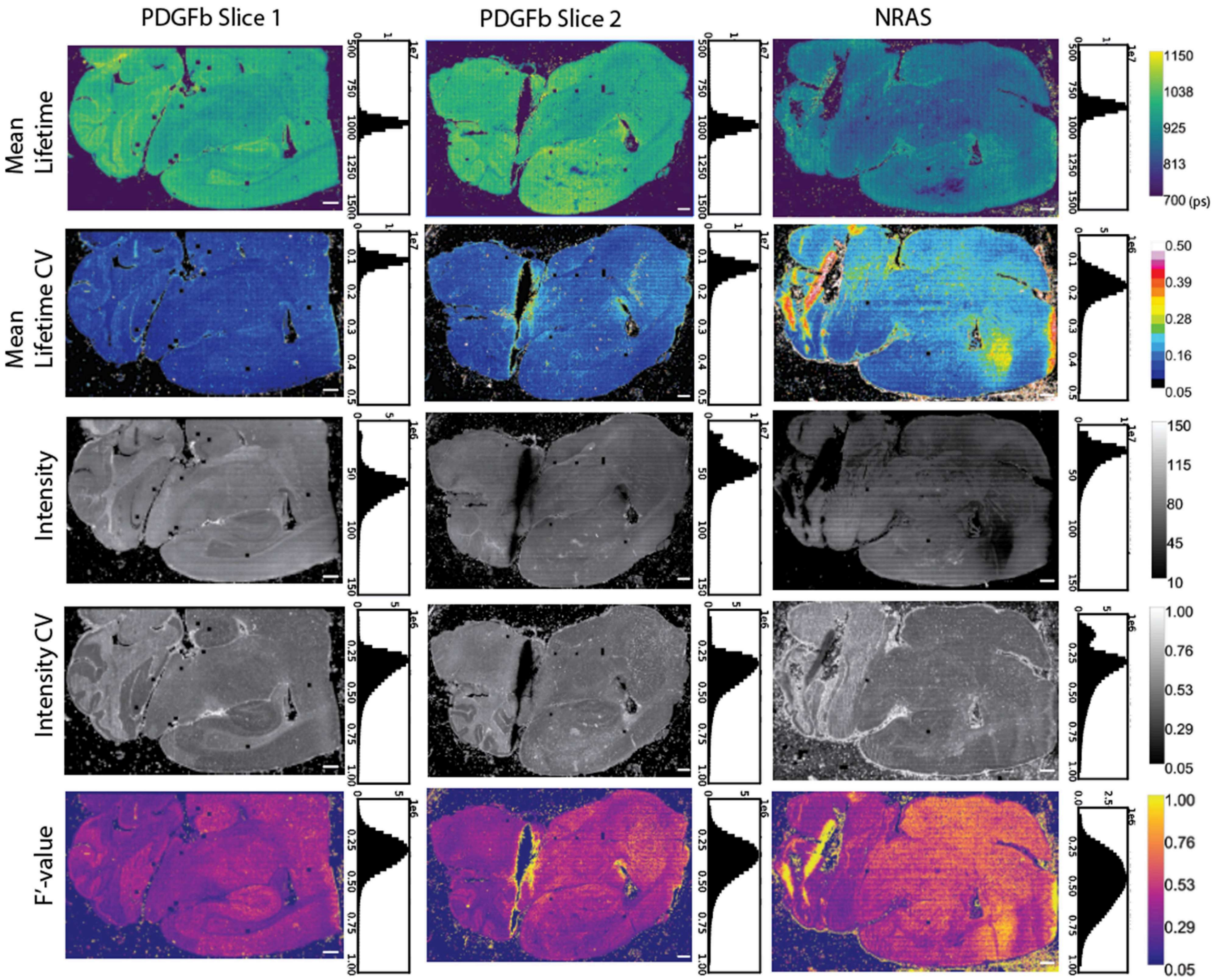

Figure 4. Representation of inter-sample variability through image displays of mean lifetime (τ), τ CV map, intensity (kernel-averaged photon counts per pixel, no binning), intensity CV map, and the corresponding F′-value (CV-metric) maps for both glioblastoma tissue types. Scale bar is 500 µm and corresponding histograms of each image are provided. The images display an overall difference in lifetime between the two types, however, local changes in intensity and F′-value help identify regions of comparable signal and SNR in each tissue.

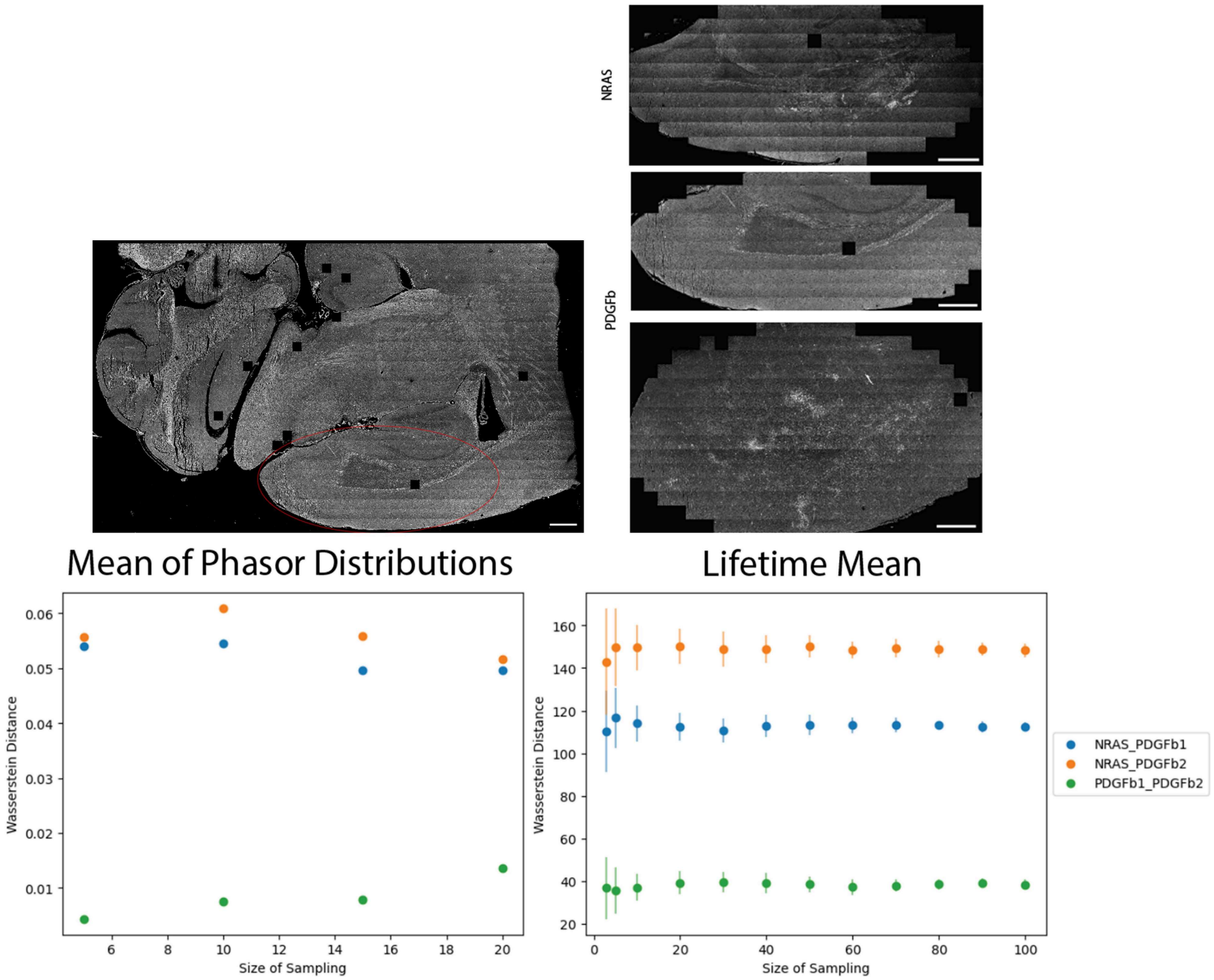

Figure 5. Higher sampling sizes of ROIs improves confidence in data separation. Top left: Approximate ROI area selected for each tissue. Top right: Actual stitched images used as a pool for *n* sample size. Bottom left: Wasserstein distance separability based on the mean of the phasor distribution for *n* images. Bottom right: Wasserstein distance separability score calculated with mean lifetime from *n* images, repeated in 30 trials. Standard deviation bars show more confidence in separation with more images. Scale bars 500 μm.

3.3. Inter-sample variability

As an example use case we examine inter-sample variability across the two glioblastoma types (figure 4). Although we see similar bulk lifetime values from each PDGFb slice and lifetime shift in the NRAS tissue (also see figure S6), the lifetime images alone do not fully summarize the data. There are large differences in intensity across the three tissues, likely due to sample variation as all three were collected with similar laser power and the same collection time. Notably, we have acquired several thousand images to capture the tissue section areas. The striping across the intensity images is in part due to the estimate of the z plane during acquisition and slight variation in focus. Areas of each sample also see changes in the F′-value, indicating a better SNR across certain parts of the image. The F′-value is calculated as the ratio of the lifetime CV and the intensity CV (see methods 2.2.2) - ideally we see both of the numbers are low and the ratio is closer to one. The CV maps of lifetime and intensity indicate specific regions of the tissue with high variability. The CV is normalized, allowing for comparisons across different sample lifetimes. The CV and F′-value maps could be used to highlight tissue morphological changes between different fluorophore populations, or identify areas of the tissue that may need to be imaged with different acquisition parameters (e.g. higher laser power). When the CV of lifetime and

intensity is combined into the F′-value metric, this value is analogous to SNR for FLIM data, and can be used to identify outlier images or comparable regions of tissue within and across a dataset. The chi-squared data for each tissue is provided and does not show significant variation, indicating a good model for the lifetime fit (figure S7). Here we see that intensity becomes a strong driver of F′-value changes in the tissues. At a large scale, we see similar trends in the F′-value for the PDGFb tissues and a shift towards higher F′-values for this NRAS tissue. With a lower intensity in large sections of the NRAS tissue, the shorter lifetimes have a higher CV, indicating we may want to consider data filters due to the change in SNR for comparison between sample types. In an ideal case, this low-intensity area could be recollected with more photons, however, it is often not practical due to resource constraints for imaging and post-processing.

As researchers often focus on sampling ROIs in a tissue area, we selected a large region of the cortex (see Supplemental figure 8) and randomly selected *n* images for analysis, (figure 5) to replicate an experimental acquisition of random areas of interest within a sample. The phasor data was computed in one trial due to slow computation, however the mean lifetime images were repeated in 30 trials. We see the increased sampling maintains a similar trend in the data (figures 5 and S8), but improves confidence in separating the lifetime distributions according to the Wasserstein distance, as noted by the smaller standard deviation bars. The Wasserstein distance can be used to denote how similar two distributions are and how much cost is associated with turning one distribution into the other [26]. Our analysis suggests that, with random sampling, small image sample sizes can still be representative of a larger tissue area for phasor data and mean lifetimes, although confidence in data separation will improve with larger sampling. In this small dataset, we observed larger differences between NRAS and PDGFb tumors than between PDGFb samples.

3.4. User interface F′-value visualization

We use the F′-value metric as a way to visualize and sort the lifetime data throughout the tissue (figure 6). The user interface which we have developed helps quickly visually this range for each sample and assist with the post-processing and data analysis by exporting binary masks of the thresholded regions. The user interface can also be used to check for outliers in a dataset and determine if any images need to be recollected or if additional data is required. The masks can be used to filter the data down to regions with similar F′-values during further analysis, including lifetime comparisons. Here we applied this to the collected data (figure 4) and used the F′-value across samples to ensure ROIs have similar SNR when comparing the data. We see the NRAS tissue has a shorter mean lifetime, indicating a trend towards more glycolysis compared to the PDGFb tissues based on the NAD(P)H measurement. Future work with larger sample sizes would be beneficial to confirm these trends, although it is similar to previous research showing mesenchymal subtypes are associated with glycolysis and proneural subtypes are associated with oxidative phosphorylation [27]. Furthermore, our lifetime analysis shows low F′-value areas have a higher variability in lifetime signal (figure 7). While we see reduced variability in the 0.25–0.4 filter and the 0.4–1 filter, it should be considered that the dark green (0.4–1) area of the NRAS tissue had a lower intensity which could skew the F′-value. To use this method, a careful understanding of the F′-value in various configurations (e.g. high photons-long lifetime versus low photons-short lifetime), and how it is influenced by sample morphology, should be closely monitored when applying the masks before further analysis. For example, the F′-value alone is not a definitive metric for a ‘good’ image as the CV for lifetime and intensity can be high in an image and the ratio is still close to one. To support examining other FLIM metrics, the user interface loads images other than F′-values so masks with various filters can be combined after export, such as a chi-squared or intensity threshold.

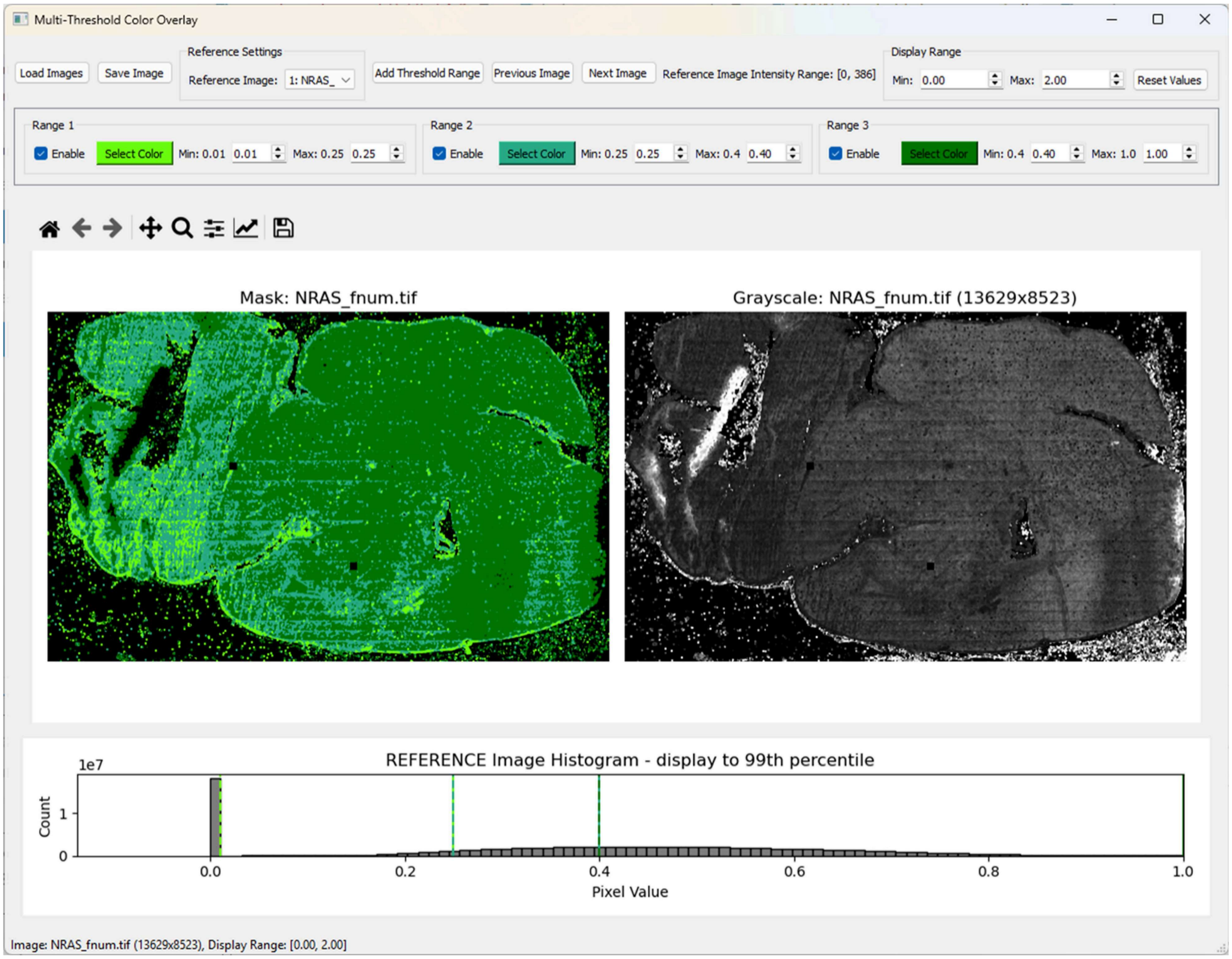

Figure 6. Display of the user interface designed for creating histogram-based image masks with an example tissue image loaded. The mask here is created based on the F′-value histogram ranges applied to the current image.

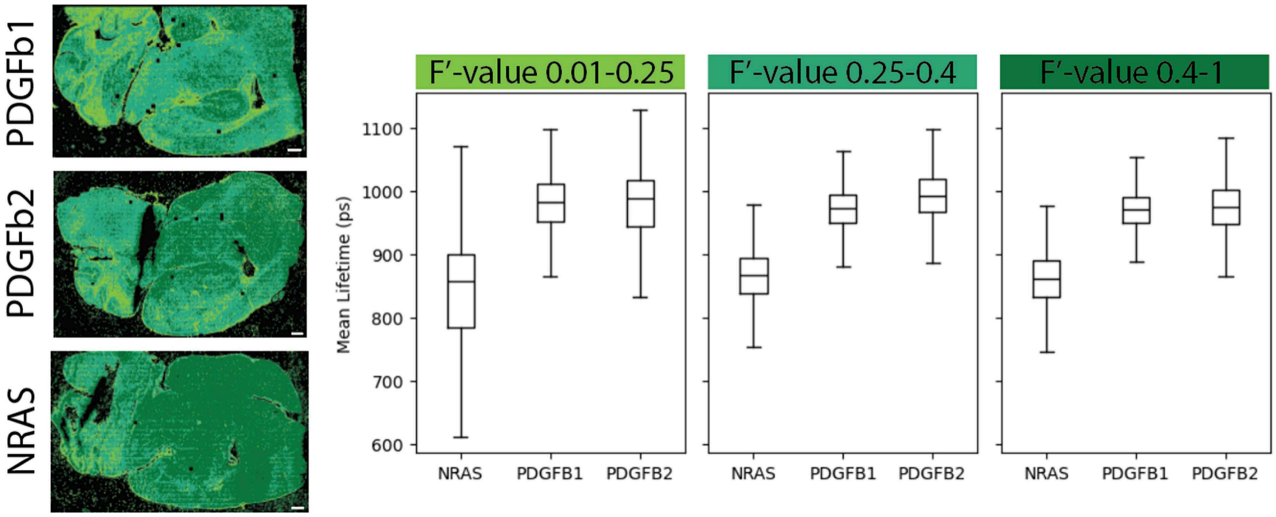

Figure 7. F′-value masks identify areas of the tissue with comparable SNR according to selected ranges. On the left, the images display F′-value thresholds as color masks, scale bar 500 μm. On the right are the respective boxplots of mean lifetime (ps) at the low, medium, and high F′-value range.

4. Discussion

Analysis of fluorescence lifetimes is normally completed through fitting models or phasor plots. We have previously demonstrated increasing variability in the lifetime measurement with longer lifetimes for single-exponential decays analyzed with both methods [9]. Here we additionally show the skew of variability using multi-component decay simulations, as often found in biological data, showing increased coordinate variability across phasor space with longer lifetimes. Our data indicates that shorter lifetimes are easier to separate if the photon counts are equal. For experimental planning of FLIM experiments, especially when extrinsic fluorophores are being used, selecting probes with separable lifetimes is critical. These simulated phasor plots can be used to help estimate expected data. Overall, we see more photons per decay curve increases the separability of fluorophore species and improves point localization. We provide the code as a tool for researchers to identify expected phasor populations based on given photon numbers and lifetime parameters.

Due to the large scale of tissues and other biological samples, microscopy imaging is often limited to comparisons using subsets of ROIs or low magnification views. Large-scale imaging of the sample at high resolutions is typically not feasible due to long-acquisition time and computational constraints, especially considering FLIM data often takes 60 s or more per image to collect sufficient signal. While this localized view of a sample can be informative, it complicates the interpretation of the data when the meaning is extracted to a larger-scale phenomena. A focus on data replication and reproducibility is the ideal way to tackle these challenges, but again this may be complex in situations with precious samples or limited resources. Here we discuss the use of the F′-value metric to help regulate the variability and SNR within a complex dataset and to develop more consistency in FLIM data analysis.

Assuming proper FLIM acquisition, regional variability in a sample is most often linked to the underlying biology as morphology changes can lead to intensity and lifetime differences across a sample (figure 3). We see local differences in intensity and lifetime which are partially dependent on the tissue structure, such as in the cortex or the cerebellum. Furthermore, due to the influence of photon number per decay curve in the confidence of a lifetime estimation, researchers must be careful to ensure similar photon levels across a dataset, even when using standardized collection parameters. While changes in photon count will not directly affect the lifetime itself, it can affect the variability shown in the measurement and therefore the confidence for statistical differentiation of the samples. The chi-squared value is the most common metric used to determine goodness-of-fit and identify outliers in a FLIM dataset, however, our data does not see the same patterns of variability in the chi-squared image as compared to the F′-value image (figure S7). In this case, a chi-squared filter alone would not be stringent enough to filter out or identify areas of high variability in the tissue. Figure 4 displays examples of the CV in intensity and lifetime as well as its effects on the F′-value in both the same tissue type (two PDGFb samples) and in another sample (NRAS). It is necessary to visualize these metrics throughout a dataset rather than relying on the lifetime values alone to confidently differentiate samples. Due to these expected changes within a given sample, we recommend thorough publishing of FLIM data and analysis metrics, including the intensity data and analysis parameters for fitting, rather than providing only the analyzed lifetime values. Researchers should use combined FLIM metrics as a whole to determine the consistency of a dataset with consideration of morphological or sample dependencies, taking into account biological changes as well as errors associated with FLIM collection.

Ideally, ROIs of the tissue areas that are compared within or between samples should have similar SNR or F′-value (figure 4) to ensure significant differences are minimally influenced by variation in lifetime or intensity, especially across small sample sizes. Based on the Wasserstein distance (figure 5), there is more confidence in the separability of the tissues at higher sample sizes, but

in this case small ROI sample sizes are also able to resolve the differences. Ensuring adequate SNR across small sample sizes is critical. By providing a visual method of interpreting the local SNR, work with ROIs can improve as researchers have fast feedback on the quality of the data and their confidence when comparing across samples (figures 6 and 7). Our python visualization tool can be used to assist the data evaluation. For example, if a group is interested in examining ROIs of the cortex, each sample image can be checked to make sure it falls within a selected SNR or F′-value range before the image is used for data analysis.

Using the F′-value and other masking parameters to create consistent datasets is useful, but does not address the direct sources of variability in FLIM. It also has the drawback of being a supervised method, which relies heavily on the judgement of a proper image. In contrast, many image analysis workflows are using semi-supervised or unsupervised machine learning algorithms to address data separation and clustering through population characteristics. These methods, such as variational autoencoders, can be utilized to assist data analysis with the benefit of having physics-informed parameters applied to data characterization. These models will begin to greatly improve FLIM analysis workflows, especially as the sources of variability are better understood.

This FLIM variability work was demonstrated and completed with fixed tissues for the pathology relevance, but also to aid in large tissue FLIM collection. However, temporal considerations for live samples should also be addressed in future work. Dynamic changes to lifetime due to biological processes in cells will continuously impact the variability associated with the lifetime measurement, and photobleaching with repeated imaging can also affect the intensity variation. As these components are often linked - more photons typically produce a more accurate decay estimation - estimating the true SNR of live samples is complex and should be examined in-depth.

5. Conclusion

In this paper we have used the F′-value to provide more comprehensive descriptors of FLIM variability arising from morphological changes and photon count differences in large-area FLIM image data. We display increased variability at longer lifetimes in phasor space, which can be addressed by using higher photon counts and understanding the placement of data. The F′-value can be used to identify areas of an image or images within a dataset that have similar amounts of variability, due to biology or FLIM collection, assisting with creating reproducible data and potentially identifying outlier areas. We also provide a python user interface to allow researchers to perform similar comparisons and visualization across their own datasets. The F′-value can be used as an additional indicator of variability within a FLIM dataset and as a tool for data evaluation, leading to more consistency in FLIM analysis.

Acknowledgments

Thanks to members of the Eliceiri, Provenzano, and Odde labs for useful discussions. Specifically, many thanks to Ghaidan Shamsan and David Largaespada for technical assistance in providing the mouse brain tumors, as well as Edward L. Evans III for help with the user interface, and Mark A. Tsuchida for help with the image acquisition code.

Data availability statement

All data that support the findings of this study are included within the article (and any supplementary files).

Funding

We acknowledge funding from NIH grants U54CA268069 (KWE, PPP and DJO) and P01CA254849 (PPP and DJO).

Ethics statement

All animal work was approved by the University of Minnesota Institutional Animal Care and Use Committee (IACUC) and all animal and experimental work followed institutional and NIH guidelines.
